# Discontinuation of Tyrosine Kinase Inhibitors in Patients with Chronic Myeloid Leukemia: a Review of the Biological Factors Associated with Treatment-Free Remission

**DOI:** 10.1007/s11912-022-01228-w

**Published:** 2022-02-10

**Authors:** Ruth Stuckey, Juan Francisco López Rodríguez, María Teresa Gómez-Casares

**Affiliations:** 1 Hematology Department, Hospital Universitario de Gran Canaria Dr. Negrín, Barranco de la Ballena s/n, Las Palmas, Spain; 2 Medical Science Department, Universidad de Las Palmas de Gran Canaria, Las Palmas, Spain

**Keywords:** Chronic myeloid leukemia, Treatment-free remission, Predictive biomarker, Leukemic stem cell, Somatic mutation, Digital PCR

## Abstract

**Purpose of Review:**

Clinical factors alone do not enable us to differentiate which patients will maintain treatment-free remission (TFR) from those who are likely to relapse. Thus, patient-specific factors must also play a role. This review will update the reader on the most recent studies presenting biological factors that can help predict tyrosine kinase inhibitor (TKI) discontinuation success.

**Recent Findings:**

Cellular and molecular factors with a suggested role in TFR include immune factors and leukemic stem cell (LSC) persistence; the BCR::ABL1 transcript type, halving time, and *BCR::ABL1* DNA and RNA positivity; as well as other molecular factors such as somatic mutations, RNA expression, and telomere length.

**Summary:**

Our review presents several biomarkers with predictive value for TFR but also highlights areas of unmet need. Future discontinuation guidelines will likely include biological factors for the personalization of TFR prediction. However, it will be important that such advances do not prevent more patients from making a TKI discontinuation attempt.

## Introduction

Tyrosine kinase inhibitors (TKIs) have revolutionized the treatment of chronic myeloid leukemia (CML), bringing the life expectancy of TKI-treated CML patients close to that of the general population [1]. However, these drugs are associated with serious side effects (including off-target toxicities such as vascular events, cytopenias, and hepatotoxicity), have a high economic cost, and can negatively impact patient quality of life [2]. For these reasons, TKI discontinuation has become a new objective in the clinical management of patients with CML and is increasingly being taken into consideration when making treatment choices [3, 4].

An estimated 40%–60% of CML patients with long-term achievement of deep molecular responses (a prerequisite for a discontinuation attempt) can successfully discontinue TKI treatment and reach treatment-free remission (TFR), in other words, continue in major molecular response (MMR, BCR::ABL1 (IS) ≤ 0.1%) after treatment is stopped [5, 6]. Criteria for TKI discontinuation include clinical factors with an effect on the probability of maintaining TFR [3, 4], encompassing the duration of TKI treatment, as well as the duration and depth of molecular response (described in greater detail in these recent reviews [7–9]). Other patient-related factors such as age and Sokal score have been suggested to be important, although their impact is still controversial (Table 1)*.*

**Table 1:** Clinical and biological factors associated with TFR

Type of factor		Variable	Evidence	Association	References
Clinical	Patient-related	**Age**	Lacking	• Some studies suggest higher TFR in older population	[92]
		**Sokal score**	Suggested	• Low Sokal score associated with better outcomes	[35, 93, 94]
		**Gender**	Lacking	• Some studies suggest association of female gender with higher TFR	[95]
	Treatment-related	**Total TKI duration**	Strong	• Favorable impact of a longer duration of therapy	[5, 28, 36, 96, 97]
		**Duration of DMR**	Strong	• Favorable impact of a longer duration of response	[5, 28, 36, 71, 98]
		**TKI resistance**	Lacking	• Decreased TFR rate but few studies have investigated this	[65, 66]
Biological	Immune-related	**CTLs**	Suggested	• Deficit in the expression of HLA class II and CT function in CML • Proliferation after TKI treatment • Low levels of CD8^+^ TCRγβ + T cells seem to be associated with relapse after TKI stop	[12•, 13, 21, 26]
		**Tregs**	Suggested	• Decrease in number with TKI treatment • Lower counts related with TFR	[12•, 14, 25]
		**pDC**	Suggested	• Lower CD86 + pDC cell ratio was found to be predictive of TFR	[12•, 22, 23]
		**MDSCs**	Suggested	• Decrease in number with TKI treatment • Lower counts related with TFR	[12•, 25]
		**NK cells**	Strong	• Proliferation with TKI treatment • Increased activating NK cells associated with maintained TFR	[12•, 19–21, 25]
		**LSC**	Evidence lacking	• LSC intrinsic factors and medullary microenvironment implicated in residual disease and a possible target for future therapeutic pathways	[6, 18]
	Transcript and molecular-related	**Type of transcript**	Conflicting	• Superior patient outcomes for e14a2 vs. e13a2 • Possible technical bias as amplification efficiency with qPCR higher for e13a2	[41–45, 46••, 47–51]
		**BCR::ABL1 DNA/RNA positivity**	Suggested	• Positivity for both DNA and RNA indicative of a higher rate of relapse when TKI was discontinued • DNA negativity in granulocytes indicator of TFR	[59•, 60, 61••, 63••, 71]
		**Rate of transcript reduction**	Suggested	• Faster decline of BCR::ABL1 transcripts in the first 3 months of TKI therapy associated with a higher probability of TFR	[52–56]
		**Somatic mutations**	Suggested	• Various polymorphisms and somatic mutations associated with TFR	[21, 65, 66, 69, 70, 72, 73]
		**RNA expression**	Suggested	• Different expression profiles for patients who maintain TFR vs. those who relapse	[82•, 83–87]
		**Telomere length**	Suggested	• Correlates with response to treatment and disease progression • Shorter length related with higher TFR	[76–79]

Among patients with similar treatment duration and response, some individuals remain in TFR while others relapse (defined as the loss of MMR) [10]. Therefore, the individual’s biological factors—which may be genetic, immune, or both—must have an impact on whether a patient retains TFR or not. However, the exact biological factors that determine the success of discontinuation remain unclear and are an active area of current research.

This review will present the current studies that are helping to increase our understanding of the biological factors (clinical and non-clinical, cellular, and molecular) that may determine TKI discontinuation success (summarized in Table 1).

## Possible Biological Factors with an Impact on Successful Discontinuation

### Immune Related

Although it has been known for a long time that immune dysfunction is relevant in the development of CML [11] (reviewed in [12•]), only recently has it become the epicenter of research efforts whose aim is to identify the mechanisms of disease development and elucidate their relationship with TFR.

The initiation of TKI treatment has been shown to change the immunological state of patients. Before TKI, patients have low expression of cells presenting leukemic-associated antigens (LAA), high myeloid-derived suppressor cell (MDSCs), and regulatory T lymphocyte (Treg) counts and present a deficit in the expression of HLA class II and cytotoxic CD8^+^ T lymphocyte (CTL) function [12•, 13]. This could facilitate tumor progression and the self-preservation of leukemic stem cells (LSC), evading the host’s development of an anti-tumoral response. In comparison, once TKI treatment is commenced, an immune reconstitution has been described, represented by an increase in the number and functionality of NK and CTL cells and a decrease in MDSCs and Tregs [14, 15].

Despite efficient TKI treatment, the persistence of leukemic stem cells (LSC) that are independent of the activity of BCR::ABL1 [16, 17] and not sensitive to TKIs [17] are likely to be responsible for relapse after TKI discontinuation. Some LSC-intrinsic factors have been implicated in the persistence of residual disease, including metabolism, autophagy, and the medullary microenvironment, and are currently being explored as putative targets for novel therapies [4, 18].

What is clear is that certain immune effector and suppressor cells have a key role in the maintenance of TFR. Here, we will briefly mention some immune cell subtypes shown to be predictive of successful TFR and strongly recommend a recent and definitive review for more information on immune dysfunction and surveillance in CML [12•]:

Innate immune system: An increased proportion of NK cells was found to be associated with longer relapse-free survival [19], an observation confirmed by the EURO-SKI, STIM, and DADI discontinuation trials, to name just a few. Furthermore, TFR was associated with increased NK cell maturity—with higher cytotoxic CD56^dim^ and memory-like CD57 NK cell populations [19, 20]—while relapse after TKI interruption was associated with low levels of CD56+ cells with low expression of CD16 and CD94/NKG2 receptors [21]. Plasmacytoid dendritic cells (pDCs) were shown by RNA sequencing (RNA-seq) to have a strong inflammatory signature [22], and lower CD86+ pDC counts were associated with TFR [23].

Immune suppressive cells: Low numbers of both monocytic myeloid-derived suppressor cells (Mo-MDSC) and FoxP3+ regulatory T cells have been associated with TFR [19, 24, 25].

Adaptive immune system: Increased numbers of CTLs, particularly TCRγβ+ T cells, were also associated with TFR [21, 26].

Taking all of these observations into account, an immune effector-suppressor score was recently developed to predict TFR success at TKI stop [25], although it may have limited applicability since most standard laboratories do not routinely measure these cell populations. Furthermore, the use of any of these immune-related biomarkers for TFR prediction will need to be tested in longitudinal studies.

One aspect that has aroused particular interest is the relationship between the patient’s immune cell composition and TKI therapy outcomes. Achievement of deep molecular responses (DMR, i.e. MR^4^ or better) was shown to be associated with high CD4^+^ T cell count, low neutrophil count, a low proportion of PD1^+^ TIM3^−^ CD8^+^ T cells, and a high response to LAAs [15, 27••]. This evidence suggests that the immune recuperation of CML patients upon TKI treatment is progressive, which is in accordance with studies that show that the global duration of TKI treatment directly influences the success of discontinuation [5, 28].

It is also clear that different TKIs have distinct immunomodulatory effects, for example, the number and function of NK cells increases during treatment with imatinib, whereas dasatinib has been shown to increase CTL and NK cell numbers [14, 29]. These differences could help to understand why DMR can be achieved with second-generation TKIs in a shorter period of time [30, 31]; thus, treatment change could accelerate eligibility for a discontinuation attempt [3]. However, the precise action of each TKI remains unclear, with conflicting results from in vitro and clinical data [12•].

Early evidence for the role of the immune response in TFR was based on observations from many years ago that interferon alpha (IFNα) could improve TKI response due to a toxic effect on the LSCs responsible for CML recurrence [32, 33] and that prior treatment with IFNα was associated with higher rates of TFR [34–37]. These observations have led to suggestions that IFNα maintenance therapy after TKI stop could be a strategy to improve TFR rates [38], an approach that is currently under investigation in the ENDURE-CML trial (https://clinicaltrials.gov/ct2/show/NCT03117816), with the IFN + TKI combination trials TIGER (nilotinib + IFN) and BOSUPEG (bosutinib + IFN) also underway. Similarly, a phase I clinical trial is exploring maintenance with lenalidomide to maintain TFR [39].

### Transcript Related

#### Type of Transcript

The close monitoring of BCR::ABL1 transcript levels by real-time quantitative PCR (RT-qPCR) is essential for patients who discontinue TKI treatment [3, 4]. However, the quantification of transcripts according to the IS [40] is only possible for the typical transcripts e13a2 (b2a2) and e14a2 (b3a2), detected in approximately 98% of CML patients [41].

Studies have long reported a superior outcome for patients expressing the e14a2 transcript compared to those expressing the e13a2 transcript, including a deeper and more rapid molecular response as well as superior progression-free and overall survival [42–44]. Moreover, some studies have suggested that patients expressing e14a2 transcripts have a higher probability of achieving TFR than those expressing e13a2 [45, 46••], although others have failed to observe statistically significant differences [47].

Importantly, a study comparing amplification of the e13a2 and e14a2 transcripts revealed that e13a2 amplification efficiency by qPCR was higher than for e14a2, which translated to an underestimation of the quantified values for e14a2 compared to e13a2 with this method [48]. This technical efficiency of amplification issue may be due to the larger size of the e14a2 transcript (with an additional 75 bp in *BCR* exon 14). In contrast, no such differences in the amplification efficiency were detected when transcripts were quantified using digital droplet PCR (ddPCR).

The molecular monitoring of rare transcripts is more complicated, as atypical fusions are detected inefficiently (or not at all) using standard RT-qPCR assays [49]. Thus, the use of bespoke RT-qPCR assays [50] or regular FISH analysis [3] is required for patient follow-up. As such, most guidelines do not recommend the discontinuation of patients with rare fusions since transcript quantification cannot be expressed on the IS for residual disease monitoring [3] but rather as an “individual molecular response” (IMR) [50], and so very few studies to date have attempted the discontinuation of patients with atypical transcripts. Nevertheless, one retrospective study of 7 patients with atypical transcripts (4 with b3a3, 2 with e19a2, and 1 patient with e8a2) reported very good results, with 6 patients retaining TFR after a median follow-up of 25 months [51].

Although controversial, TKI discontinuation could be deemed safe for CML patients with atypical BCR::ABL1 transcripts as long as transcript-specific regular monitoring can be carried out by a specialized laboratory, and particularly if a highly sensitive technique, such as ddPCR, is available.

#### Rate of Reduction of BCR::ABL1 Transcripts

An optimal early response to TKI therapy is an important determinant for long-term outcome in CML [52, 53], as the values of BCR::ABL1 transcripts and the kinetics of their descent in the first trimester (also commonly referred to as halving time) are predictive of deep MR thereafter [54–56].

Recently, Shanmuganathan et al. observed that a faster decline of BCR::ABL1 transcripts in the first 3 months of TKI therapy was also associated with a higher probability of achieving TFR, with 80% of patients achieving TFR if the transcript halving time was less than 9.4 days compared to just 4% if the halving time was more than 21.9 days [46••]. Therefore, the kinetics of transcript reduction in the first trimester of commencing TKI therapy is important to predict TKI response and predictive for TFR as well.

#### Profundity of Molecular Response

Although RT-qPCR is the gold-standard method for the molecular monitoring of BCR::ABL1 transcripts [57], ddPCR detects these molecules with a higher sensitivity than RT-qPCR, permitting the detection of just 1 BCR::ABL1 molecule in 100⋅000 cells [58]. A study by Nicolini et al. suggested that this increased sensitivity can help to discriminate patients who are more likely to relapse after TKI discontinuation [59•]. The authors used ddPCR to analyze the RNA samples of 175 patients from the STIM2 study who had discontinued imatinib and had undetectable transcripts according to the RT-qPCR technique. The median transcript value, used as a cut-off, was 0.0026%, converted to 0.0023%^IS^. Patients with a ddPCR value below 0.0023%^IS^ had a two-fold lower risk of relapse than those with values above this threshold [59•].

Nevertheless, the DESTINY trial reported a thought-provoking result. The TKI de-escalation trial compared two groups of patients whose TKI treatment was reduced to half the standard dose for 12 months prior to discontinuation: a group who discontinued while in stable MMR versus a group who stopped in stable MR^4^. The authors reported a TFR rate of 36% for the MMR group and 72% for the MR^4^ group [60]. Although 64% of patients in the MMR group relapsed after TKI discontinuation, the authors reflect that 36% did maintain TFR and that perhaps this group of patients would not necessarily be eligible to make a discontinuation attempt in many centers.

In conclusion, the absolute quantification of BCR::ABL1 transcripts, both typical and atypical, with the use of ddPCR may be useful for the monitoring of residual disease in patients attempting a TKI discontinuation and could help prevent the inclusion of transcript-related bias in patient outcomes. Nevertheless, despite the superior sensitivity of the ddPCR technique, particularly when samples are analyzed in triplicate or quadruplicate [58], conversion factors would need to be agreed by the CML community to permit the standardization of transcripts on the IS before laboratories could consider replacing their method of choice for molecular monitoring.

#### Presence of BCR::ABL1 RNA and DNA

As previously mentioned, many patients in DMR with undetectable transcripts by qRT-PCR do have residual BCR::ABL1 RNA if a more sensitive technique is used [59•], as well as detectable *BCR::ABL1* DNA [61••]. One recent paper developed a simple traffic light approach to predict patients likely to relapse, based on the detection of residual BCR::ABL1 RNA and DNA using ddPCR prior to TKI discontinuation (i.e., while in DMR). The positivity for both molecules was indicative of a higher rate of relapse when TKI was discontinued (20% were relapse-free after 18 months), while DNA negativity but RNA positivity was associated with an intermediate risk (57% were relapse-free after 18 months), and DNA and RNA negativity was associated with higher rates of TFR (80% were relapse-free after 18 months) [61••]. The authors suggested that *BCR::ABL1* DNA detection by ddPCR prior to a TKI discontinuation attempt could help to predict which patients are likely to relapse.

In a second similar study, the separation and isolation of specific cell populations were used to increase the sensitivity of *BCR::ABL1* molecular analysis [62] in order to evaluate the lineage of residual CML cells in TFR [63••]. Of the 20 patients in TFR for at least 1 year, 18 had detectable *BCR::ABL1* DNA in the total leukocyte population (analyzed by a sensitive nested qPCR technique), predominantly in B cells, but also measurable in T cells and NK cells. In contrast, *BCR::ABL1* molecules were never observed in the granulocyte populations extracted from patients in TFR. When analyzed in the sample taken at TKI stop, 100% of the patients with granulocyte-positive for DNA relapsed within the first 3 months [63••]. Thus, the authors propose that the detection of *BCR::ABL1*-positive granulocytes can predict relapse. However, it remains to be seen whether this method, requiring the prior separation of cell populations [64], will be implementable in standard hematology laboratories.

### Other Molecular Factors

#### BCR::ABL1 Kinase Domain Mutations

In earlier versions of the NCCN and ELN guidelines, TKI resistance was among the exclusion criteria for a TKI discontinuation attempt. However, TKI resistance is no longer excluded in the 2021 NCCN guidelines on discontinuation [4], whereas the 2020 ELN guidelines state that TKI discontinuation is “allowed” if tolerance is the only reason for changing TKI; thus, resistant patients would be excluded although not explicitly [3].

Indeed, very few studies have reported cases of patients with a previous history of *BCR::ABL1* kinase domain mutations that have attempted discontinuation. Of the 60 patients recruited in the STOP 2G-TKI trial, designed to evaluate the discontinuation of second-generation TKIs, 13 had a suboptimal response to imatinib, 4 of which were due to an *BCR::ABL1* kinase domain mutation that conferred imatinib resistance [65]. In univariate analysis, suboptimal response to imatinib or TKI resistance was a baseline factor associated with molecular relapse, although the impact of TKI resistant mutations alone was not reported.

However, in one small-scale study, of the 10 patients who discontinued TKI treatment (9 of which had detectable mutations, including T315I), 5 maintained TFR [66]. Therefore, whether patients with a previously reported *BCR::ABL1* kinase domain mutation can maintain TFR and thus safely make a TKI discontinuation attempt remains to be confirmed by larger studies.

#### Somatic Mutations and Polymorphisms

The development of new technologies such as next-generation sequencing (NGS) has led to significant advances in our understanding of the molecular pathogenesis of hematological neoplasms. In CML, various mutations associated with response to TKI and/or progression to accelerated phase or blast crisis have been reported, suggesting a role for additional mutations besides *BCR::ABL1* in the evolution of CML disease [67•]. One theory on the biological difference between patients with an otherwise similar clinical profile who achieve TFR and those who relapse is based on the presence of polymorphisms or the accumulation (or disappearance) of somatic mutations during the course of TKI treatment associated with maintaining remission following TKI discontinuation.

It is known that during TKI treatment, CML patients accumulate or lose mutations, which influence the patient’s response to TKI [68]*.* For example, mutations emerged in the first 6–12 months after initiating TKI in 37% of CML patients, with good responders having a lower frequency of acquired mutations. Specifically, mutations in *TP53* were associated with poor TKI response.

The gain or loss of a mutation might favor activation of the immune system, which could be sufficient to maintain the molecular response when the TKI is retired. For example, associations have been reported between polymorphisms in the HLA (human leukocyte antigens) genes and patients achieving TFR, with polymorphisms in HLA-A*02:01, *11:01, or *24:02 significantly associated with TFR [69] and HLA-E*01:03 associated with relapse [21]. Similarly, a study of polymorphisms in the killer immunoglobulin-like receptor (KIR) genes and patients achieving TFR support this hypothesis [70]. In the 36 CML patients with a MR^4.5^ who discontinued TKI treatment, a significantly higher number of patients with the KIR A/A haplotype in homozygosis were in TFR compared with those with haplotype B/x (86% vs. 46%), with a later study confirming the association of the B/x haplotype with relapse after discontinuation [70]. Nevertheless, the EURO-SKI study did not observe differences in TFR according to the KIR haplotype [71].

Wider genetic studies have identified other variants with a putative role in maintaining TFR. In one such study, Shen et al. reported that the SNP rs139130389 in the folate receptor 3 (*FOLR3*) gene is an indicator of TFR. CML cells harboring this SNP had greater proliferative and colony-forming ability due to increased mitochondrial activity [72]. Similarly, a 2016 study sequenced the exome of 6 CML patients who discontinued TKI treatment (3 who relapsed and 3 in TFR) and identified variants in the genes *CYP1B1*, *ALPK2*, and *IRF1* in patients who relapsed and a variant in *PARP9* in the group with TFR [73].

Whether new mutations are acquired in the LSC of CML patients on TKI treatment remains to be elucidated [74]. However, one recent study, presented in the 2020 EHA meeting, showed that the DNA damage response (DDR) could be a potential biomarker of molecular relapse, finding increased DDR in CD45 + CD34 + CD38-CD26 + LSCs compared to normal hematopoyetic stem cells with the CD45 + CD34 + CD38-CD26-immunophenotype, with patients in TFR found to have less DNA damage at diagnosis [75].

#### Telomere Length

Numerous early studies showed that the telomere length of CML patients (adjusted for patient age, “age-adjusted TL”) correlates with response to treatment and disease progression [76] (reviewed in [77]). A later study showed that age-adjusted TL was also associated with TFR, with a higher proportion of CML patients with shorter telomeres maintaining TFR after TKI discontinuation than patients with longer telomeres (79% vs. 31%) [78]. Interestingly, telomere shortening was shown to occur in LSC but not in hematopoietic stem cells (HSC) in CML patients [79] and to be associated with genetic instability (reviewed in [80]) and an inflammatory profile [81].

#### Expression Changes

Other molecular factors with a possible role in the success of a TKI discontinuation attempt include the patient’s transcription profile. For example, the level of mRNA expression of the *ABCG2* efflux transporter was an independent predictor of TFR after TKI discontinuation. Specifically, CML patients with an ABCG2/GUSB transcript level below 4.5% had a significantly higher 12-month TFR rate than those with high ABCG2 expression (72% vs. 47%) [82•].

Moreover, as a result of the development of RNA-seq and single cell RNA-seq, global studies have reported a distinct gene expression profile for patients who have an suboptimal response to TKI, including a signature enriched for stem cell phenotype, reduced immune response, and cell cycle [83] as well as the existence of a Lin^−^CD34^+^CD38^−/low^CD45RA^−^cKIT^−^CD26^+^ LSC population [84] that persists during TKI therapy. The detection of a LSC population with such an expression profile (i.e., increased proliferation-associated and reduced quiescence-associated gene expression) could in the future be used as an indicator that the patient may not be a suitable candidate for TKI discontinuation [85].

A proof-of-principle study presented in this year’s John Goldman ESH congress described how differentially expressed genes can be identified between patients who maintain TFR versus those who relapse [86], supporting the hypothesis that a distinct gene expression profile could help predict patients who obtain TFR. However, the RNA-seq studies reported to date are preliminary and have not yet established a signature profile using transcriptome data to reliably predict patients who will retain TFR from those likely to relapse.

## Future Perspectives

Although promising, the application of RNA-seq expression profiles as a predictive tool for patients who will retain TFR still needs to be established. In future expression studies, it will also be interesting to study the impact of microRNAs (miRNA)—non-codifying RNA molecules of 19–25 nts that have a posttranscriptional role and can inhibit translation and/or reduce the stability of the mRNA molecule by binding to the 3′ UTR of mRNAs—on TKI response and TFR. For instance, the expression of miR-215 was significantly lower in patients in TFR versus those on imatinib with undetectable disease [87]. Whether differences exist in the expression levels of other miRNA molecules in patients who achieve TFR and those who relapse, as well as the impact of epigenetic changes in TFR remain to be elucidated. One hypothesis is that BCR::ABL1-induced epigenetic changes may not be fully reversed by TKI and thus could persist [88].

Although many biological factors have been identified, to date, little is known about the intra- and inter-patient differences that exist. In addition, the impact of the specific TKI (or series of TKI) taken by the patient on biological factors such as somatic mutations and gene expression profiles and patient outcomes, including TFR, remains to be determined. In this respect, there is hope among the international CML community that the HARMONY Plus/ELN/iCMLf project will help elucidate novel biological factors associated with TFR. The big data project’s objectives include “to create a better understanding of the clonal evolution of CML to improve patient management” and “to help understand additional mutations that would allow a larger number of patients to remain in therapy-free remission after treatment discontinuation” [89].

Importantly, a small group of patients remain who cannot reach deep remissions even with prolonged treatment. If current guidelines are followed meticulously, these patients might never be candidates for discontinuation. Thus, an important area of reflection for the CML community to consider is whether the introduction of more stringent inclusion criteria, such as deeper molecular responses prior to TKI discontinuation, is likely to prevent a significant number of patients from attempting to stop treatment (who might retain TFR).

Improvements to the molecular responses of these patients also constitute an area of unmet need. A promising strategy for these patients could be IFN combination treatment, since 73% of patients who discontinued while in MMR were in TFR after a median follow-up of 7.9 years as a result of this approach [38]. Other studies aimed at improving the molecular response of this group of patients might identify mechanisms of LSC persistence and/or prospective therapeutic targets that could benefit all CML patients. Besides interferon, many novel therapies are currently under development for use in combination with TKI, including immune-checkpoint inhibitors and even CAR-T therapy to target the persistence of LSC [12•, 90, 91].

## Conclusions

**Fig. 1:**
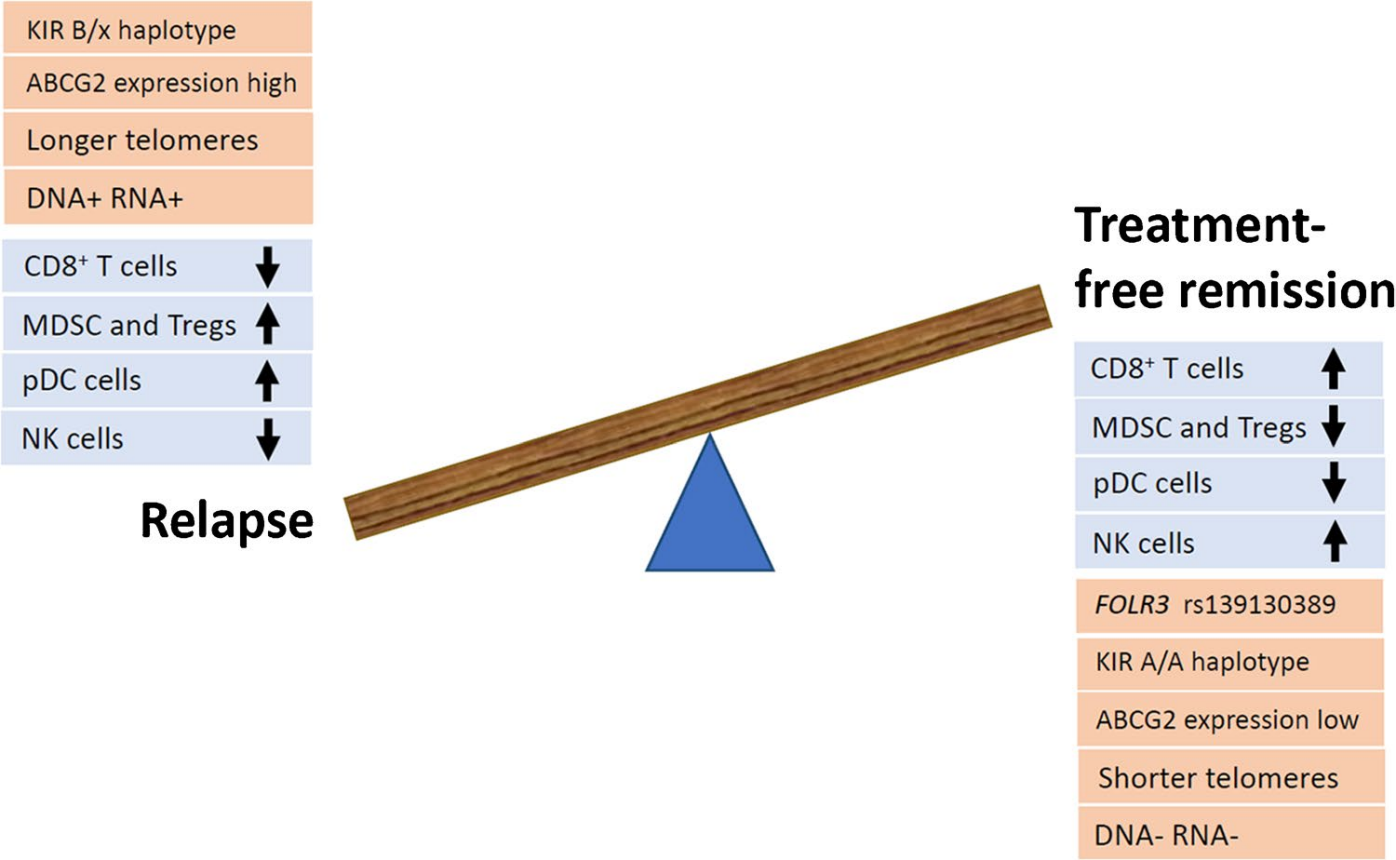
Schematic representation of factors reported to be associated with TFR or molecular relapse, including immune cell subtypes (in blue) and other molecular factors (in orange)

Clinical factors alone do not enable the accurate stratification of patients with CML according to their risk of relapse. Several biological factors have been presented in this review as being predictive for TFR in CML (Fig. 1). However, there is a real clinical need to continue to study both patients who maintain TFR and those who suffer relapse in order to understand the mechanisms of LSC persistence and the cellular and molecular factors influencing TFR.

The future of TKI discontinuation is to personalize the prediction of TFR by integrating clinical factors with emerging biomarkers to inform clinicians on the candidates with a greater probability of success. Greater knowledge of the biological factors associated with TFR could lead to the development of more precise and individual discontinuation criteria, but it is important that such criteria allow the majority of CML patients to benefit from a TKI discontinuation attempt.
